# Economic impact of Ebola virus disease outbreak on an extractive firm: a case study

**DOI:** 10.14324/111.444/ucloe.000007

**Published:** 2020-05-13

**Authors:** Hisham Tariq, David Tresco Emes, Yebeen Ysabelle Boo, Alexander Light, Zia Sadique, Mishal Khan, Alan Knight, Osman Dar, Logan Manikam

**Affiliations:** 1 THINKLab, The University of Salford, Maxwell Building, The Crescent, 7^th^ Floor, Salford M5 4WT, UK; 2 Centre of Disaster Resilience, The University of Salford, The Crescent, Salford M5 4WT, UK; 3 Oxford Department of International Development and Department of Economics, University of Oxford, Oxford OX1 3TB, UK; 4 UCL Institute of Epidemiology and Healthcare, University College London, Gower Street, London WC1E 6BT, UK; 5 Aceso Global Health Consultants Ltd, 3 Abbey Terrace, London SE2 9EY, UK; 6 Population, Policy and Practice, UCL Great Ormond Street Institute of Child Health, 30 Guildford Street, London WC1N 1EH, UK; 7 Bedfordshire NHS Foundation Trust, Bedford, UK; 8 Department for Global Health and Development, London School of Hygiene and Tropical Medicine, 15–17 Tavistock Place, London WC1H 9SH, UK; 9 ArcelorMittal, 7^th^ Floor, Berkeley Square House, Berkeley Square, London W1J 6DA, UK; 10 Chatham House, 10 St. James’ Square, St. James’s, London SW1Y 4LE, UK

**Keywords:** Ebola, epidemic, economics, health economics, Liberia, Africa, sanitation, health, and the environment, environmental economics

## Abstract

**Purpose:**

The recent Ebola virus disease (EVD) epidemic was one of the most severe public health emergencies in modern times. The economic impact of epidemics has mostly been analysed at the macroeconomic level. Conversely, we aimed to estimate the economic costs of preventive measures of the epidemic to an extractive firm, ArcelorMittal (AM), using data in the epidemic region from March 2014 to December 2015. AM is the world’s largest steel producer and is particularly important in West Africa, where the extractive industry is economically crucial.

**Methods:**

Qualitative methods, in-depth interviews (IDIs) and focus group discussions (FGDs), were used to investigate the events and channels of impact of the epidemic on the firm, as perceived by employees and contractors. Quantitative data regarding these costs were also collected. Retrospective cost analysis estimated the actual cost of preventive methods adopted.

**Results:**

Most respondents indicated the largest cost impact was suspension of the Phase II expansion, a series of projects designed to increase iron ore production in Liberia. The next largest cost was the preventive measures adopted to counter disease spread. Total costs incurred for adopting preventive measures were USD 10.58–11.11 million. The overall direct costs of preventive measures adopted within the fence, meaning within the physical boundary of the firm’s sites, shared 30–31% of the total costs incurred. The share of external donations supporting humanitarian response was 11–12% of the total costs, followed by 7–12% of relational costs.

**Conclusions:**

The firm’s response during the EVD epidemic focussed on its employees and operations, which was later expanded to the wider community and then in supporting the international humanitarian response.

## Introduction

The Ebola virus disease (EVD) epidemic of 2014 in West Africa was unprecedented, leading to a public health emergency on a new scale. The morbidity and mortality impact of the 2014 EVD epidemic was far larger than all previous EVD epidemics combined [1], and the overall economic impact of the epidemic for the most Ebola-affected countries including Liberia, Guinea and Sierra Leone, was estimated at USD 2.2 billion in gross domestic product (GDP) losses [2]. The economic impact of epidemics has frequently been reported at the macroeconomic level, that is, an impact affecting the aggregate economy of a country. However, there has been relatively little investigation into the effects epidemics can have at the microeconomic level on individual market participants, such as those in the extractive industry [3]. This industry comprises firms that extract raw materials from the earth, and is very important to the economies of West African countries in GDP terms, where EVD epidemics are more likely [4], although some have argued that the industry has yet to fully contribute to the developmental goals of these states, such as in stronger healthcare systems of the local communities where their extractive activities are mainly concentrated [5].

Extractive projects put workers at high risk of exposure to pathogens such as the Ebola virus. These projects are frequently associated with increased contact between wildlife, humans and domestic animals, a major risk factor for the emergence of infectious disease [6]. The extractive industry, through its operations, necessarily brings about changes in the social and natural environments such as advancing into new uninhabited areas where operations like exploration, extraction/mining activities and developing transportation networks in these remote areas lead to increasing contact with wildlife. This places a significant burden on local ecosystems, and as local economic systems emerge to support increasing worker populations, opportunities increase for infections like EVD to breach the species barriers between animals and humans. The EVD crisis therefore posed a major economic threat to mining operations and future mining investments in the region. This threat was compounded by changes in market conditions, including a decrease in the global price of iron ore.

The Ebola virus epidemic reportedly began in West Africa during late 2013. It started in Guinea and spread at an alarming rate, quickly involving Liberia, Sierra Leone, Nigeria, Senegal and Mali [6]. This is the first EVD epidemic to reach epidemic proportions; previous epidemics were localised and were brought under control within a few weeks using methods such as effective reporting, contact tracing and quarantine [7]. On August 8, 2014 the World Health Organization (WHO) declared the epidemic to be a ‘public health emergency of international concern’ and later the most severe acute public health emergency seen in modern times [8–10]. The epidemic was eventually halted, with transmission is now effectively contained within the region. Since 2015 only isolated cases have been reported in Sierra Leone, Guinea and Liberia [11–14].

Countries in West Africa are rich in many mineral deposits including alumina, bauxite, cement, diamonds, gold, mineral sands and iron ore [15, 16]. The iron ore industry in particular has played a key role in the economic growth of Liberia and Sierra Leone, contributing heavily to their economies over the last few years [2, 15]. Mining, a critical sector, had been affected by the EVD epidemic directly through expansion delays, lack of new investment, absence of technical expatriate staff and perception issues that have made it difficult for mining companies to raise necessary capital [17]. This had been exacerbated by a concurrent decline in the global price of iron ore. Due to the importance of mineral deposits to the economies of these countries, the extractive sector is an important industry to consider within the larger economic impact of the EVD epidemic in affected regions. How these regions are affected by the epidemic, and how they respond to the epidemic by adopting preventive measures, has potential economic consequence on the extractive industry and overall economies in the Ebola-affected region. One major firm within the extractive industry is ArcelorMittal (AM), a multinational company headquartered in Luxembourg. Being the world’s largest steel producer [18], the firm had planned a series of projects in Liberia known as Phase II, worth USD 1.7 billion. These projects aimed to expand iron ore production for AM from 5.2 million to 15 million tons per annum. AM was significantly involved in developing EVD outbreak infrastructure in the region and was recognised by the Clinton Global Initiative for its swift and collaborative response [19].

In the context of the 2014 EVD epidemic, it is important to document the actions of a multinational firm in their response to the epidemic, the various channels of impact of EVD epidemic, as perceived by their staff working during the period, on the operations of the firm in the extractive sector and to estimate the costs of the preventive measures that were put in place. Additionally, documenting the actions and measures taken during this period can possibly help to identify longer term benefits (or otherwise) to the firm, as well as their employees, of continuing operations during the lockdown of the country where effective travel between locations was limited. This study was therefore designed to estimate the direct and indirect costs of preventive measures of the EVD epidemic to AM and its subsidiary ArcelorMittal Liberia (AML), using data from a case study based in the outbreak region. Documenting the actions of one of the few firms that continued to operate during the EVD epidemic period may provide some benefit to other firms and business looking to build organisational resilience to epidemics in the future. Therefore, this study aims to qualitatively analyse the perception of staff and contractors of the firm to the EVD epidemic, its impact on the firm in terms of actions taken (or not taken), on supply chains, on the operational cost of the firm, and then to estimate the actual costs incurred by the firm for preventive measures adopted during the EVD epidemic period.

## Methods and approaches

### Study setting and design

The case study was based on AM and its subsidiary AML. The study applied a mixed methods approach to assess the firm level impact of the EVD epidemic on the case study firm with an emphasis on quantifying the total costs of the preventive measures taken by the firm during the EVD epidemic period from March 2014 till December 2015. The study used qualitative methods to investigate and map the sequence of events and the various direct and indirect impacts of the epidemic on the firm, as perceived by its employees and contractors. The quantitative data on the direct and indirect costs of the EVD epidemic to the firm was extracted from interviews, financial documents and other materials provided by AM and its employees. This data was further validated in group sessions with relevant AM personnel.

Informed by the qualitative analysis and the quantitative data collected from the various departments across AML including the finance department of AML, as well as crosschecking with eternal sources, the study estimated the actual cost of the preventive measures both ‘within the fence’, which indicates expenditures made within the physical boundaries of the firm’s sites, and ‘outside the fence’, which indicates expenditures made on actions that primarily take place outside of the firm’s physical boundaries, that the firm had adopted during the epidemic period.

### Data collection and data management

The research study used in-depth interviews (IDIs) and focus group discussions (FGDs) to collect qualitative data as part of the case study approach. In order to facilitate and guide IDIs and FGDs, guidance notes were prepared in advance (Appendices A and B). IDIs were conducted in English and lasted approximately 45–60 min. FGDs were conducted in English and lasted almost an hour, consisting of five to seven participants. Participants in IDIs and FGDs were asked for consent before digitally recording their responses, where consent was not given detailed notes were made. These recordings were than transcribed by one member of the research team and the transcripts were then checked by other team members for accuracy. These transcripts were then all entered in Nvivo version 10, manufactured by QSR International (Melbourne, Australia), for further analysis. Nvivo is a commercially available software for qualitative analysis, no funding was received to use this software. All paper and soft copies of field notes, audio files, contact summary forms, enrolment forms, consent forms and any other notes were kept securely. The digital formats of IDIs and FGDs were anonymised, password protected and saved in a secured location. 

Through the IDIs, respondents were asked to list what they believed were the most critical systems impacted within the firm by the EVD epidemic and, if applicable to them, were asked to provide information on how the cost structure within the firm may have been affected accordingly. Questions were asked about the preventive measures taken by the firm, their knowledge and opinion about the implementation of those measures and their understanding of how it may or may not have impacted their work. If known to them, the costs of those measures were also asked about. Detailed data on the cost components identified in the qualitative study were sought from relevant departments of the firm.

The FGDs were conducted at the end after all interviews were completed, where they were used to check if the key findings from the IDIs were valid and correct. The questions for the FGDs were formulated by the research team after the preliminary analysis of the first set of IDIs and were emailed to respondents in advance to ensure that they were aware of the type of questions being explored. Subsequently, the transcripts were checked by the other study team members to ensure that the questions and format of the FGDs were followed as designed.

### Analysis

#### Qualitative analysis

The study used interview data to identify major sources through which the epidemic impacted the different aspects of the firm’s operation and consequent operational costs. Respondents were asked to list and rank what, in their opinion, were the three major cost impacts of the EVD epidemic on the firm’s operations. It was reported that there were opportunity costs of staff time devoted to dealing with the epidemic. Relational costs of the EVD epidemic, the costs indirectly arising from the impact of the EVD on the operations of the firm, were investigated using data from qualitative interviews. The interviews were transcribed individually into Microsoft Word documents using ScribePro and analysed thematically using content analysis to derive the main concepts about the perceptions of the employees as well as key information about the decision-making within the firm.

#### Analysis of quantitative data released by the firm

A retrospective cost analysis was performed from the firm’s perspective using the financial information extracted from the interviews, FGDs and the financial documentation provided by AM. This entailed a detailed look at the cost and expenditures data shared by the various departments of the firm – tabulating them chronologically and cross checking between sources to ensure consistency and accuracy. The policies, their costs and impacts were validated in further group sessions with participants, who had knowledge about financial expenditures, at the end of the study. Similarly, the opportunity costs of the relational items were quantified using the wage data of the firm obtained from both qualitative interviews and quantitative data.

#### Items, outputs and costs

The financial information collected from the firm detailed the specific dollar costs of all items purchased during the epidemic period – both those items purchased directly for the preparedness, mitigation and response phases and, also, the items purchased in routine operations during this period to compare with data from the previous year’s purchases. This comparison helped the team develop a better understanding of dollar expenditures towards additional activities and towards routine total costs. Additionally, total production or output level data was also obtained from the relevant department for several years so that a comparison to similar periods in the previous years could be made. Finally, total cost data that covered the above two items for the firm were also obtained for the period of the epidemic.

#### Sources of information

The primary source of all quantitative data was the Accounts and Payroll section of AML. The data sets used were given to the team in Excel sheets and data were shared for the 2014 epidemic period as well as data from the previous 2 years for comparison. The data shared were shared in two batches, one before the fieldwork began and one post the fieldwork, although additional clarifications and some figures were shared on direct request throughout the study period whenever gaps or missing information were perceived. Needless to say, the process was not easy and securing the data was a difficult task especially as the study took place just after the epidemic period and the firm was still operating under EVD epidemic conditions.

#### Sampling

Table 1 shows the number and composition of the sample of respondents interviewed across different locations and occupational categories within the firm. Although a cross section of employees was taken in the study, more interviews were done with those in management and finance than other departments due to their role in decision-making within the firm, their knowledge about costing information and their access to financial data. Most respondents were from senior management and had considerable industry experience.

**Table 1.** Sample size and composition.

Occupational category	London	Liberia	Total	Remarks
Senior Management^a^	7	7	14	Workshop conducted with six ex-executives from London
Professional, Administrative and Technical Management (PATM)^b^	1	5	6	In Liberia: IDIs with seven expatriates and nine localsOne FGD with expat contractors (seven participants)
Skilled^c^		4	4	One FGD with Liberian employees (six participants)
Total	8	16	24	Skilled and unskilled

a Recruited from the following departments: Communications, Corporate Responsibility, Finance, Human Resources, Supply Chain/Logistics, Health & Security and Government Relations.

b Recruited from the following departments: Administration, Risk Management, Environment, Health & Safety, Supply Chain, Security and Transport.

c Recruited from the following departments: Estate, Maintenance, Port, Mine, Rail, Security and Transport.

## Results

The results from the quantitative assessment from the data provided by the firm and validated by the qualitative interviews on the perception of staff and employees of AM, along with their experience of the epidemic period are given below. The results from both these analyses were then subsequently validated by the FGDs.

### Quantitative data

#### Cost impacts

The quantitative data indicated firstly that the single largest cost of the EVD epidemic in 2014 to the firm was from the range of preventive measures put into place in the firm’s concession areas and raising awareness in the adjacent community. Secondly, was the in-kind donations of priority materials and direct support to national and international engagement in the health and humanitarian crisis that was verified by internal and external sources. Thirdly, Ebola-related construction costs constituted the next highest dollar value expenditure, followed finally by the additional salary paid to workers as hazard pay during the epidemic period, and evacuation of non-essential staff (NES). There were other costs, including the lost productivity from workers’ engagement with health and safety (H&S) measures during the epidemic period. The total preventive costs of the epidemic incurred by the mining firm were mainly driven by direct costs and relational or productivity costs as reported in Table 2. The total preventive costs of the epidemic were in the range of 10.58 million USD to 11.11 million US. The range arises from uncertainty in only one element of the costs, the relational costs.

**Table 2.** Costs of preventive measures.

Costs	Estimated USD (in millions)
Within the fence preventive measures	3.29
External donations	1.27
Construction related	1.56
Salary	2.41
Evacuation of non-essential staff	1.27
Relational	0.78–1.30
**Total costs**	**10.58–11.11**

The overall direct costs of preventive measures adopted within the fence, shared 30–31% of the total costs incurred (Table 2). The share of external donations supporting the humanitarian response was 11–12% of the total costs, followed by 7–12% of the relational costs. Construction-related costs comprised 14–15%, salary comprised 23%, and evacuation of NES comprised 11–12% of total costs.

#### Preventive measures

**Figure 1:**
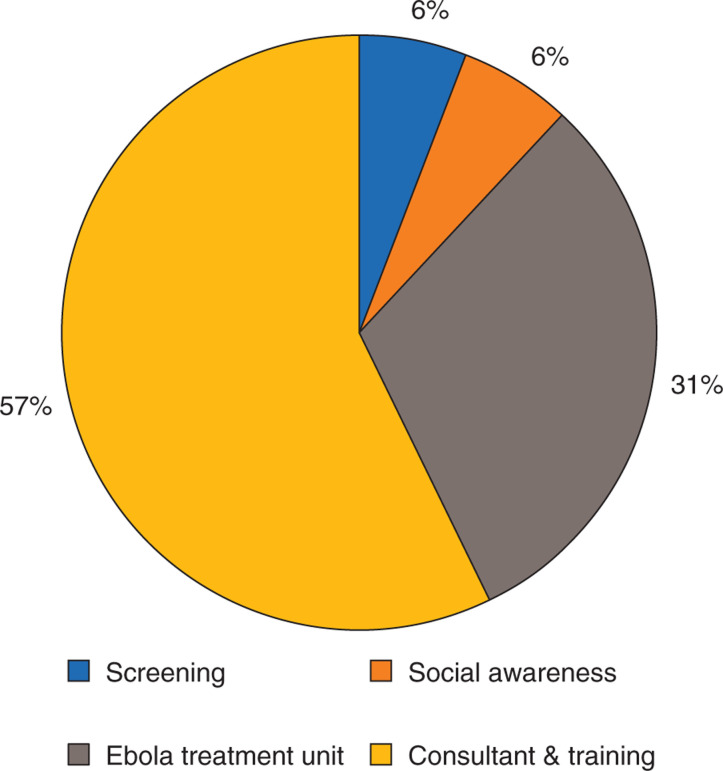
Percentage distribution of costs related to preventive actions adopted on site and adjacent community.

Fifty-seven percent of the preventive costs were incurred from payment to consultants (International SOS, a medical and travel security services firm) and training for putting the security and safety measures in place (Fig. 1). The costs of building an Ebola Treatment Unit (ETU) for treating suspected or infected cases were 31% of the total preventive costs. Costs related to screening everyone entering the site and building social awareness in the adjacent community was 12% of the total preventive costs.

#### Donations and direct support to the health and humanitarian crisis

**Figure 2:**
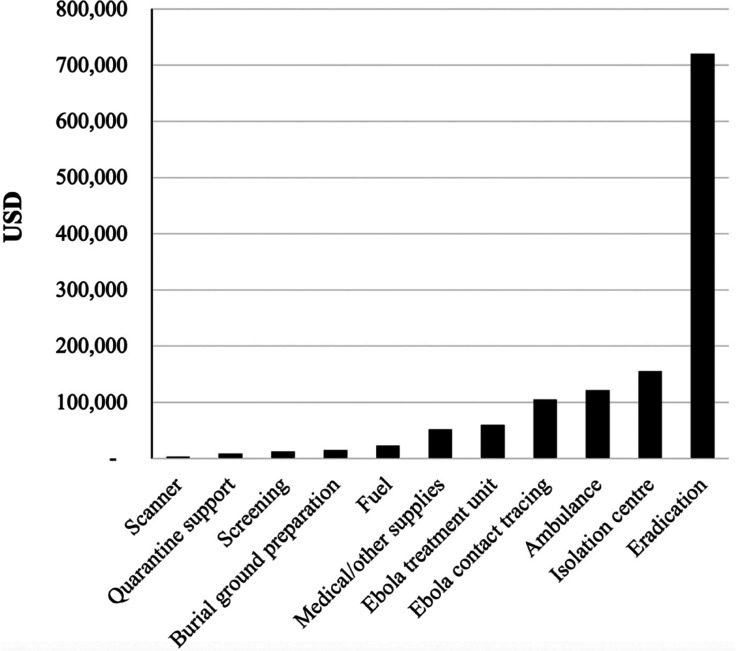
Costs of external support by activities.

The mining firm made donations to the international public health and humanitarian response communities to support the prevention and treatment of Ebola. The costs related to donations and support to the external Ebola response was approximately USD 1.27 million. The major share of the external support was for supporting the response towards the eradication of EVD, followed by building three isolation centres, donations towards the ambulance services, contract tracing, machinery and capacity to construct external ETUs, as well as other essential medical supplies as shown in Fig. 2. Donations also included supplying fuel, preparing burial grounds and preventive actions (screening, quarantine support and installing a scanner at the airport).

The firm provided external support to many Ebola initiatives around the community. The largest share of the external support was provided to support the activities of the Red Cross (56%), followed by providing funds for the county’s regional task forces formed at the local government level to deal with the epidemic (28%), hospitals (11%), county/township services not covered under the taskforce roles (4%) and other beneficiaries including the airport, the police and other government departments (1.2%).

#### Human resource policies – hazard pay and evacuation of non-essential staff

The firm paid hazard and incentive payments to the workforce to help maintain a stable supply of workers during the epidemic period. The costs of additional salary payments during the epidemic totalled USD 2.41 million. The cost of evacuation of expats and NES was USD 1.27 million.

#### Relational costs

Based on the distribution of the workforce and the hourly wage rate across employment categories (Table 3), the costs of lost productivity were in the range of USD 0.78–1.30 million.

**Table 3.** Distribution of workforce and wage rate.

Employment category	% of total work-force^a^	Hourly wages ($)
Senior management	1.2	150.0^a^
PATM	14.6	6.0^b^
Skilled	72.4	4.7^c^
Unskilled	11.8	3.7^d^

PATM, Professional, Administrative and Technical Management.

a Obtained from qualitative interviews.

b Calculated from average monthly salary of employees working in following departments: Communications, Corporate Responsibility, Finance, Human Resources, Information Technology, Legal, School and Technical Services.

c Calculated from average monthly salary of employees working in following departments: Administration, Environment, Health & Safety and Supply Chain.

d Calculated from average monthly salary of employees working in following departments: Estate, Maintenance, Port, Mine, Rail, Security and Transport.

#### Ebola-related construction costs

These additional construction-related costs totalled USD 1.56 million.

### Findings from the qualitative data

The presentation of findings from the qualitative IDIs and FGDs is detailed next and helps provide context and background to the results in the previous section as well the general perceptions of the workforce during the epidemic period.

#### Cost impacts

**Figure 3:**
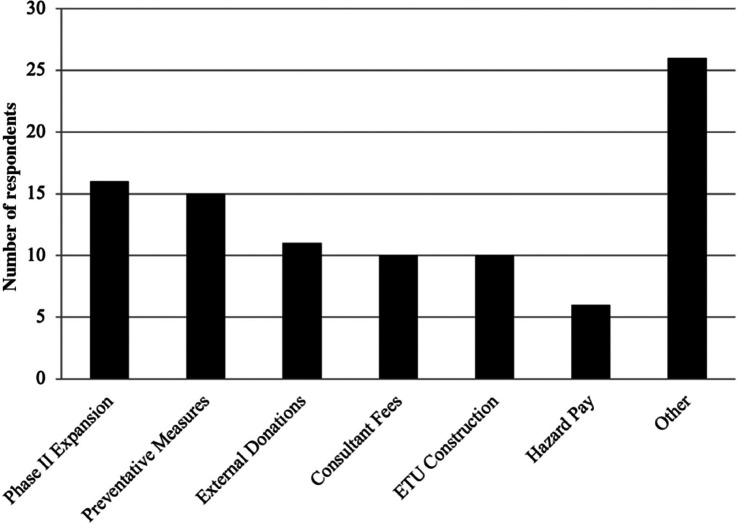
Major cost impacts as perceived by respondents.

Fig. 3 shows the major cost impacts of the EVD epidemic as perceived by the respondents. The majority of the respondents (n = 16/24, 66.6%) indicated that the suspension of the Phase II expansion was the largest cost impact on the firm. The next largest perceived cost impact (n = 15/24, 62.5%) was the preventive measures adopted by the firm to counter the spread of Ebola followed by the external donations mentioned as the third largest cost impact (n = 11/24, 46%), although this could be due to the proportionately larger number of senior management interviewed who would be more concerned about reporting such external donations. Respondents also indicated several other sources of impacts which include the impact associated with administrative issues, loss of efficiency due to temporary redundancies and hazard pay.

#### Phase II expansion

Though not a preventive measure, the suspension of the Phase II expansion was cited the most times (n = 16/24, 66.6%) by respondents. The project has been placed on hold till further notice by the firm, more due to the international market price of iron ore than the aftereffects of the Ebola epidemic. The resultant loss in overall production and revenue has significantly impacted both the firm and Liberia itself. The assessment of what the total costs of this suspension will be is beyond the scope of the study. Respondents mentioned that one reason for the suspension of Phase II in 2014 may have been due to airlines ceasing services to Monrovia, the capital city of Liberia:

*The [Phase II] construction was impacted because our contractors had to leave…some of them…because of Ebola fear…they did not leave because of Ebola but because of the restrictions put on travel…and they did not want to be hemmed in.*

The mining firm decided to delay and eventually (late 2015 – early 2016) to temporarily suspend the expansion which may have been due to the tough international market conditions for iron ore in that year (2015) in addition to the impact of the EVD epidemic.

*The 2014 dates [of force majeure] were more driven by the contractors themselves saying…you know we are pulling out…this [risk] is not acceptable to us – 2015 was more I suppose driven by the firm in the sense that we were actually calling the suspension to the project…but one kind of fell after the other…it was a bit of a domino…but certainly you know if Ebola had not come in theory we would have…you know those months we lost we would have had contractors on the ground and they would have been constructing and we would have been further down the road then we are now.*

#### A quick response, consultant costs and training

The major cost impacts that were reported and pointed out by the respondents were part of the package of preventive measures that the firm adopted over the epidemic period. Fourteen of the 24 respondents (n = 14/24, 58%) had the opinion that one of the key factors that separated the firm from other extractive industry members was the proactive nature of the firm in seeking information from a world-renowned health expert in the field relatively early in the process. The health expert spent three crucial weeks of April 2014 in Liberia and advised the firm on preventive measures while collaborating with International SOS, in developing their medical response to the epidemic. This intervention was important for AM both as a tool for the internal communication of risks within the organisation and for providing insight into strategies to safeguard their employees, the concession and the communities around it.

#### Social awareness campaign and programmes

Risk communication materials developed during this initial phase (in April–May 2014) helped the firm in Liberia to distribute large amounts of printed material across their concessions initially targeting employees, their families and then the wider community at large (June onwards). Some respondents (n = 7/24, 29%) indicated that there were additional roles they had to conduct during the epidemic period. These roles included conducting a social awareness campaign on Ebola, delivering hand washing buckets and sanitizers and other activities within these communities. This was particularly for those communities inside or in close proximity to the concessions.

#### Screening and fencing, and Ebola treatment units

Temperature screening and access control were some of the first steps recommended by the experts and these were quickly implemented by the firm in Liberia throughout the concessions where the firm operated. Fencing around the key operational sites in the concessions was completed by the end of June and by beginning of July temperature screening and hand washing stations were operating at all entry and exit points of the fenced zones in the firm’s concessions. These steps were quickly absorbed into the H&S culture already prevalent in the firm. Multinational extractive firms in general must follow international H&S standards and the routine training, certification and adherence to these industry standards may have contributed to the swift implementation of temperature screening and fencing protocosl throughout its operations. The strict observance of temperature screening protocols was reported positively by several respondents (n = 8/24, 33%) as the primary reason for staying Ebola-free within the fence in the concessions throughout the epidemic period, whereas a large majority (n = 19/24, 79%) indicated it played an important role in maintaining operations. Respondents gave several examples from memory of incidents of suspected Ebola cases being refused entry that later became confirmed EVD cases. During the epidemic period in late November 2014, AML built and maintained two separate ETUs at considerable cost. A large number of respondents (n = 10/24, 41%), perceived the ETUs construction as the single most expensive expenditure. The two new ETU buildings were specially designed and equipped to handle three confirmed Ebola patients each (total six) and were developed to stabilise Ebola patients until they could be evacuated to their country of origin. 

#### Human resource policies – hazard pay and evacuation of non-essential staff

Several human resource policies were enacted during the epidemic that had significant cost implications for the firm. Two specific policies were indicated in the interviews, the hazard pay policy and the NES policy implemented throughout August and September 2014. The hazard pay policy was costly because of the number of employees that qualified for it. In effect, all those who were classified as essential workers would receive it. In addition to the hazard pay policy, those employees who were considered as non-essential were sent home and were also paid a salary although at a reduced rate. NES were asked to work from home where they continued to support the firm’s operations remotely. Another major cost impact was the evacuation flights arranged for ex-pats and NES. The evacuation flights were triggered after most international airlines cancelled their flights to and from Monrovia, Liberia in August 2014. A number of respondents (n = 6/24, 25%) indicated that together the hazard pay and the NES policies had the largest cost impact of the epidemic on the firm’s operations.

#### Relational costs and the emergency management team

Some respondents (n = 5/24, 20%) indicated that there was an administrative productivity loss (relational costs) due to the preoccupation with EVD management and, also, as a direct result of some of the preventive measures taken. In early 2014, the firm began reviewing and updating emergency management plans in Liberia in case of major security incidents or natural disasters. The firm had decided at that stage to develop emergency management teams (EMTs) in Liberia as part of a crisis management infrastructure within the firm. These teams consisted of senior management and other concerned staff, as dictated by the needs of the crisis, and would be enacted on an emergency by emergency basis. These EMTs played a crucial role in responding to the EVD epidemic as there was a crisis management structure which the firm in Liberia could then build on and link to a central EMT located in London.

*Even before the outbreak… in Liberia…so, for example, if we had a major security incident or natural disaster…anything…we could manage it effectively from the company side. So, we put in place what was called an emergency management team… really all they consisted of was a lot of the head of departments from the CEO on to Health & Safety, Security, Communications and Medical.*

The EMTs connected daily for several hours a day for the duration of the outbreak and played a key role in the decision-making process, with regards to what preventive measures were to be taken and when. These meetings of senior management and staff for several hours a day during the peak months of the epidemic had significant cost implications.

The qualitative interviews suggest that on average senior management spent 1.5 h per day during the peak Ebola period (August–November 2014), followed by 1 h daily during the off-peak epidemic period (December 2014–June 2015) and 0.2 h daily during the super off-peak epidemic period (July–December 2015), on these meeting. The terms peak, off-peak and super off-peak were used by the senior management team in the EMT based in London to describe the crisis period from their point of view in terms of how much time they allocated to the meetings. In the base case, only the staff time of the senior management is costed to value relational costs. The daily hours spent by senior management on Ebola-related activities during peak and off-peak period varied between the lower (1 h during peak, 0.5 h during off peak) and upper limit (2 h during peak, 1 h during off peak) as indicated in the qualitative interviews. We have also evaluated the time costs of all other employment categories to predict possible relational costs when an epidemic affects staff time and productivity across the board. It was inferred from statements by operational staff that all other workers spent on average 0.2 h per day on Ebola-related activities over the epidemic period (August 2014 to December 2015).

#### Ebola-related construction costs

During the EVD epidemic period, the firm also incurred additional construction costs for mining activities while maintaining the security and safety of its workforce when considering the risks related to Ebola transmission. These costs were incurred from constructing gates, installing washing stations and building fencing as safety measures adopted to fight Ebola.

#### Other costs items such as alternate logistics, stockpiles and the supply chain

One of the crucial impacts of any disaster-related disruptions is on the supply chain of the firm. The epidemic caused considerable issues with logistics and this was confirmed in the interviews with employees across several departments within the firm. A few respondents (n = 3/24, 12.5%) also indicated that some of the extra costs and bottlenecks in operations were a direct result of issues in the supply chain and logistics. This is especially true for a firm in the extractive industry working in conditions like Liberia where most supply items, if not all, are imported from other countries. However, this could not be documented through quantitative means due to the unavailability of data from the concerned department. One of the policies that could be documented from other departments was the stockpiling of several items required for the implementation of key preventative measures and steps like masks, personal protective equipment (PPE) clothing, temperature screening equipment, maintaining hand washing stations and alcohol-based hand sanitizers. The costs for those items have been included in the preventative measures’ expenditure section where appropriate rather than here.

Furthermore, the interviews probed whether there were additional costs of the epidemic from the shutdown, transport costs, insurance payments and supply chain items attributable to the EVD epidemic period. The responses of key staff of the firm suggested no additional costs were incurred from any other items in addition to those already included in this analysis.

## Discussion

The IDIs and FGDs provided the team with an understanding of the perception of employees and contractors of the firm regarding the chain of events during the epidemic. They also inform the team of the different areas of expenditure costs incurred by the firm for preventive measures adopted to stop the epidemic from entering its workforce and operational sites. The quantitative data provided by the firm helped match and compare those perceptions with the actual expenditures incurred. The qualitative results show that despite initial gaps in knowledge and awareness of emerging infectious diseases (EIDs) like Ebola, AML was able to rapidly access expertise and put into place a number of preventative measures that primarily focussed on inside the fence risk mitigation that incurred additional operational costs. The responses indicated that AML had the capacity for early detection and was flexible enough to respond quickly to the situation by changing practices and allocating the required funds for its implementation. The ability to quickly adapt infection control measures and to internalise them into existing H&S mechanisms meant AML was better prepared in June 2014 when the epidemic entered the urban areas of Liberia than it was in March 2014 at the outset of the epidemic. The level of preparedness, and to certain extent the quality of mitigation measures adopted by the firm, was documented in the study through mixed methods allowing the research team to analyse the additional cost impacts that were incurred by the firm during the epidemic period.

### Cost impacts

The magnitude of the actual costs incurred by the firm largely conformed to the perceived costs identified by the respondents in the qualitative study. The actual cost of preventive measures and reduced productivity incurred by the firm was in the range of USD 10.58–11.11 million (Table 2). This range is attributable to partial data availability, as well as different perspectives within the firm on lost productivity and, also the expenditures on addressing productivity over the period. Estimates on productivity loss are difficult to obtain accurately especially during a complex event like the EVD epidemic period and it must be noted that the team had difficulty in obtaining the financial data in its entirety. The main sources of actual cost impact as indicated by the quantitative data were (a) preventive measures adopted in the firm’s concession areas and raising awareness in the adjacent community, (b) in-kind donations of priority materials and direct support to national and international engagement in the health and humanitarian crisis, (c) Ebola-related construction costs, (d) additional salary paid to workers as hazard pay during the epidemic period, and evacuation of NES.

Accordingly, the largest cost was generated from preventive health outlays and other containment measures implemented in the mining concession and in the community. The second largest costs were incurred from additional salary payments and the evacuation of NES, followed by Ebola-related direct construction costs, external support towards Liberia’s efforts to contain, treat and eradicate Ebola, and reduced productivity due to the EVD epidemic period.

The respondents in the qualitative interviews identified the Phase II expansion as the largest cost impact of the EVD epidemic period on the firm, followed by preventive measures, external donations, consultant fees, ETU construction and hazard pay. As a consequence of the suspension of Phase II, many contractors declared force majeure, when unforeseeable circumstances prevent a contract from being fulfilled, resulting in contractors pulling out of Liberia in August and September of 2014. Although the EVD epidemic may have been responsible for the series of events that led up to this declaration, the general market prices of iron ore and other considerations also played a role. In the quantitative costing, the researchers were unable to estimate the costs associated with the Phase II expansion. However, for the other items listed, the actual costs incurred largely conformed to the perceived costs. It also needs to be highlighted that the low iron ore prices over the period likely added to the uncertainties stemming from the EVD epidemic on business continuity and expansion of iron ore mining.

### Minimising disruption privately and publicly

The study shows that there was a system in place in AML for the early monitoring of threats such as disease epidemics. Despite this, Ebola was identified as a potential EID risk only when it was confirmed in Guinea in March 2014. By this time, Ebola had already spread to Liberia. For organisations to be resilient they need as much lead time as possible before a disruption, in order to develop and implement measures that can help prevent or mitigate the impacts of a disruption on its business activities. This is especially true for EIDs that can spread unnoticed in a human or animal population for a significant period of time before being detected.

Interviewees felt that one of the factors for the continued operations of the firm in Liberia during the EVD epidemic was the role attributed to communications in its corporate culture [20]. Effective communication plays a role throughout the risk management process especially when there is uncertainty in outcomes [21]. In accordance, our qualitative data indicates that AML effectively used risk communication in the implementation of preventive measures at different levels ranging from community social awareness programmes to industry collaboration in the form of the Ebola Private Sector Mobilisation Group (EPSMG) and its campaign for a coordinated international response to the EVD epidemic. The EPSMG, initiated by AM, had participated in international advocacy for a global response to the Ebola epidemic at the UN and other forums, and also contributed to the mobilisation of in-country private sector resources to support humanitarian and healthcare efforts [22]. We recommend that firms develop training programmes in crisis coordination for communication departments at both local and international levels that will improve the ability of firms in the extractive industry to respond to a disruption. Inclusion of AM communication staff in EMTs at the earliest stage is an example of what role effective communication can play in reducing fears of employees in the initial stages of an outbreak.

Private firms in the extractive industry also typically have operations in remote locations, like the border areas of Nimba County in Liberia, and can therefore play a vital role in the early detection of EIDs if connected to local health systems. This is in the interest of both the public and private sectors to increase preparation time for mitigation strategies that can limit the extent of the impact of the disruption [5]. The implementation of an EID early warning system would ensure that disruptions to business continuity from EIDs could be minimised. This could only be done if these extractive firms are integrated into the local health systems. This requires active public–private collaboration on sharing information towards developing an effective early warning system and consequent control measures.

### Supporting the community

**Figure 4:**
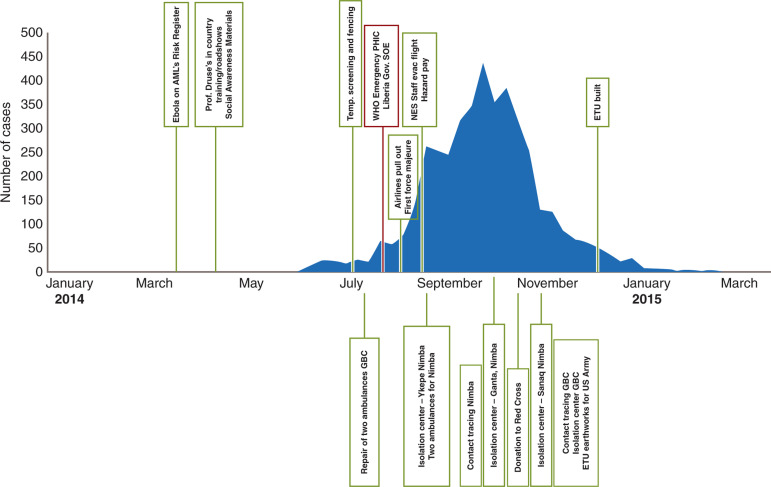
Ebola cases per week and the chronology of preventive measures and external support. GBC = Grand Bassa County; SOE = State of Emergency.

The timeline of AM’s response demonstrates that the firm was engaged in prevention, building and strengthening an EVD epidemic control infrastructure, for example, Ebola screening mechanisms and ETUs (Fig. 4). These developments occurred within the concessions and, to a certain extent, in the wider community. These developments also occurred at a time when the firm’s contribution to the epidemic response was extremely important, well before the international community’s response. The example of the EPSMG is given and was responsible for considerable community mobilisation activities and coordination among key stakeholders.

To put these expenditures on preventive measures and external donations in context to the epidemic timeline, Fig. 4 describes the chronology of when and where these expenditures were made as indicated in the interviews and quantitative data and Table 4 can be used as a legend for Fig. 4. The quick response by the firm in bringing in expertise (April 2014), within a week of putting Ebola as an EID on the risk register (end of March 2014), to help increase disease understanding is seen here as the first step towards developing and adopting a medical approach to the EVD epidemic.

**Table 4.** Timeline of events and actions taken by the firm.

No.	Approximate date	Event
1	Mid-March 2014	Ebola placed on AML’s Risk Register
2	April 2014	Prof. Duse (Infectious Disease Expert) invited to conduct risk assessments and trainings of staff
3	July	Fencing completed around main sites and temperature screening begins
4	July	Repair of two ambulances for local authority
5	July 17^th^	WHO declares emergency
6	7^th^ August	Liberian Government declares state of emergency
7	August	Airlines flight cancelations, first contractor declares force majeure
8	August–September	NES evacuation, beginning of hazard pay policy
9	September	Isolation Centre established in Ykepe, Nimba. 2 ambulances donated
10	September	Contact tracing for employees and community in Nimba started
11	September	Isolation centre established in Ganta, Nimba
12	November	Major donation to the Red Cross
13	November	Isolation centre established in Sanqa, Nimba
14	November	Contact tracing and isolation centre in Grand Bassa County
15	November–December	Earthworks done for US Army field ETUs
16	November–December	Two ETUs established for AML employees use

Etu, Ebola treatment unit; NES, non-essential staff.

Most of the respondents (n = 18/24, 75%) indicated that by the early adoption of recommended measures, like social awareness campaigns and temperature screening, the firm was reacting proactively regardless of the mortality and morbidity rates prevalent in Liberia at the time. The timeline of the firm’s response also shows that when the number of cases per week increased in June and July 2014, the firm in Liberia already had systems in place to continue monitoring its workforce and maintain its production. This commitment to be operational allowed it to be in a position to help the communities it was based in to combat the outbreak by contributing to the building of critical EVD epidemic control infrastructures, such as isolation centers and contact tracing teams, when they were needed most at the peak of the outbreak well before the international community’s response.

The timeline of response shown in Fig. 4 and Table 4 illustrates how the firm started by adopting timely preventive measures to protect its employees and operations. The success of the firm’s response in maintaining the site Ebola-free led it to expand its support to the humanitarian response in partnership with government and nongovernment organisations.

### Recommendations

There are several recommendations that can be made following this case study. Firstly, due to the importance of effective communication in the risk management process, we recommend that firms train their communications department to manage crisis coordination, both internally and externally to the communities they operate in. Risk communication vertically with government and local communities as well as horizontally with other private sector actors to form partnerships and coalitions, like EPSMG, can also contribute to operational resilience. 

Formulating such partnerships can allow private firms like AM to adopt wider early warning systems to monitor for critical events such as disease outbreak, with particular emphasis on being able to identify the potential impact of EIDs before it is too late. Integration of such a system into the local community and health system would also minimise the public impact of an epidemic. This requires significant collaboration and information sharing between public and private sectors in order to create such a system. Being able to act early and proactively allows firms not only to protect their own operations but also allows firms to commence and support their humanitarian response earlier and more productively.

### Strengths and limitations

This study’s strengths are largely related to its practical applicability in the industry. The study was set in the real-world mining firm context. The views of the experienced mining staff have strengthened the study by providing a balanced and representative view of how an epidemic can affect mining operations, and how a future crisis could be handled. The study was able to report on direct costs on preventive and mitigation measures incurred by the firm over the course of the epidemic.

This study also has several limitations. First, the study was conducted while the epidemic was continuing and AM was experiencing economic downturn, not only because of the epidemic but also due to falling commodity prices in the international market. Second, this study was designed to capture the effect of epidemic on both direct and indirect costs, especially the effect on supply chain items and future expansion projects, but it was not possible to obtain sufficiently detailed data to estimate such costs. Third, the qualitative and system analysis was limited by the availability of the key respondents during the study’s timeframe. These limitations stemmed largely from a high turnover of finance office and senior management staff over the course of the study, as well as a large number of redundancies, particularly in the finance division, which the company had to incur at the end of 2015.

## Conclusions

Our study found that extractive companies operating in outbreak-prone areas should introduce crisis communication training and support the creation of an early warning system for events such as EID epidemics. Importantly, such a system should be integrated into local community and health regimes in order to minimise the public and business continuity related impacts of an epidemic and limit potentially significant financial losses.

The cost incurred by the mining firm for adopting preventive measures during the 2014–2015 EVD epidemic was in the range of USD 10.58 million to USD 11.11 million. The response of the mining firm during the EVD epidemic was focussed on its employees and its operations, which was then expanded to the wider community and then in supporting the international humanitarian response. This was important to building and strengthening the Ebola response infrastructure of Liberia to make a concerted effort to fight the epidemic. There are several recommendations that can be made to private firms following this case study. This includes introducing crisis communication training and the creation of an early warning system for events such as EID epidemics. Importantly, such a system should be integrated into local community and health regimes in order to minimise the public impact of an epidemic. Due to a paucity of studies examining the macroeconomic and especially microeconomic impact of Ebola, further research would help strengthen understanding of the economic impact of endemics and how firms and economies can best manage epidemics.

## Data Availability

The datasets generated during and/or analysed during the current study are available from the corresponding author on reasonable request.

## Appendix A Interview guide for in-depth interviews.

**Table:**

Specific dimensions/topics	Questions	Suggested probes
Introduction/background	Please indicate what is your designation/department that you belong to and describe the nature of your role in the company as well as your main responsibilities and duties.	How many countries or sites do you manage? Is the company centralised or is decision-making devolved to the sites? Did your roles and responsibilities change in any way during the outbreak?
	How long have you worked in this company?	
	How long have you worked in the mining industry?	
	Did you work in the industry during the current outbreak period?	
	Where exactly and for how long?	
Risk and vulnerability	Can you describe any past experiences of disease outbreaks or illnesses in your mines in Liberia or other mining sites where you have worked?	What happened? What kinds of situations make these diseases more or less likely?
	How serious are these diseases for the company and local communities? Please give examples of their impacts.	
Systems affected by outbreak	Can you please list, to the best of your knowledge, which aspects of the mining operations were most affected during the outbreak? (List and rank)	Production Mining capacity Human Resources Health & Safety Why do you think this was the most affected?
Production	Can you please describe how production was affected during the outbreak? (List and rank if more than one way)	Compare to normal operations previous to the outbreak How was the production rate affected? Production goals? Daily/weekly/monthly data? How were inventory levels affected? Did this significantly affect order rates/order fulfilments?
	What were the added challenges of operating in an outbreak environment	
Factors of production (INPUTS)	How were the costs of production affected?	Of local inputs Of inputs being brought in from abroad Rental/repairs
	How were supply chains of inputs affected?	Any critical blockages that were affected for key inputs Petroleum/chemical products, etc.
Transportation and shipping (logistics)	How was transportation of iron ore affected during the outbreak?	Freight costs From sites to inventory/warehousing site Trade restrictions/border crossings, etc.
	How was the shipping rate affected, if at all?	
	Other aspects of logistics that affected mining operations during the outbreak	
Mining capacity	Can you please describe how in your understanding the mining capacity, or expansion, was affected by the outbreak? (List and rank if more than one way)	How were expansion goals/planned capacity additions affected? How was exploration affected, if at all?
	In your opinion, what effect would the out-break have on the ability of ArcelorMittal (AM) to attract future investment for mining capacity expansion?	In Liberia In West Africa In areas more susceptible to EIDs
	Capital expenditures	
	What role do foreign subcontractors play in mining capacity expansion?	i.e., any up gradation of plant machinery, equipment and/or other capital intensive expenditures affected
Human resources	What effect did the out-break have on the human resources available to AM and its mining operations?	Number of workers/level of absenteeism What major reasons for absenteeism? transport, fear, taking care of relatives
	How did you/your firm mitigate it/cope?	
	Was productivity compromised? If so, then at what levels and how?	Hiring additional workers Training
	Were there any changes in the decision making structure of your firm during the outbreak – creation of new roles/departments, etc.?	Increasing workloads/more overtime Financial incentives Productivity
	Change in standard operating procedures?	Skilled vs. unskilled Domestic vs. foreign Senior management productivity/outbreak response/workloads Time allocation being affected vs. normal Changing roles/shortages of key personnel For special areas/locations For special types of activities
H&S systems	Can you please describe how health and safety has been affected, if at all, by the current outbreak? (List and rank if more than one way)	Compared to pre-outbreak period Management commitment to safety/Time taken to respond
	How has this affected your personal commitment to safety?	Living under outbreak conditions
	How might have both these factors affected the incident rate of Ebola infections?	Risky behaviour Rate of other Incidents
	Can you describe any ways you have heard of (or been personally involved in) for preventing diseases that come from animals.	Are there any measures that can be taken at the mine itself to avoid out-breaks? Training activities Screening Temp checking
	Are any of these preventive approaches currently being used in your mining areas, in Liberia and elsewhere?	Costs Logistics Skills Manpower Equipment
	Are there any issues around:	
	Why or why not?	
	What health services or facilities does AM provide on-site for its employees?	
	What additional health services or facilities has AM been able to provide during this outbreak?	
	During the ongoing out-break has this preventive been scaled up? If so then by how much	
	How effective do you feel are the preventive measures taken by AM?	
	How have these measures affected your morale and of the employees working in outbreak areas?	

EID, emerging infectious diseases; H&S, Health & Safety.

## Appendix B Guide for focus group discussions.

**Table:**

Specific dimensions/topics	Questions	Suggested probes
Introduction/background	Please indicate what are your designations/departments that you belong to and describe the nature of your role in the company as well as your main responsibilities and duties. How long have you worked in this company? How long have you worked in the mining industry? Did you work in the industry during the current outbreak period? Where exactly and for how long?	How many countries or sites do you manage? Is the company centralised or is decision-making devolved to the sites? Did your roles and responsibilities change in any way during the outbreak?
Risk and vulnerability	Can you describe any past experiences of disease outbreaks or illnesses in your mines in Liberia or other mining sites where you have worked? How serious are these diseases for the company and local communities? Please give examples of their impacts.	What happened? What kinds of situations make these diseases more or less likely?
Risk perception	How likely is it that there might be an incident of a worker infected with Ebola in the next year	In general (London) Local site (Liberia) Magnitude question try to get answer in a scale out of 100
	How serious would it be an employee or a worker to get infected by Ebola in the next year	Elaborate as much as possible by using ‘why’ when getting a response from the respondent
	How likely do you think it is that an employee or worker will get infected by Ebola in the next year compared to other firms working in the same area	
	Do you think that people in general are informed and can take actions to prevent getting Ebola?	Locals Families of employees
Current outbreak risk	How many incidents have there been of workers from your firm getting infected during this current outbreak?	General outbreak Locality (if in Liberia)
	How severe is this outbreak (compared to any previous ones you may have experienced)?	
	What was/is the probability of an employee being infected in this outbreak? A close family member?	Locals Families of employees Subcontractors/foreign workers
	How confident are you that workers in your firm can prevent getting infected by Ebola?	Employees in other sectors Government commitment to safety of citizens
	How effective has the government been in addressing the current outbreak?	Time taken for response Response/actions taken
Knowledge and sources of information	How much do you know about the Ebola virus?	
	What are the main sources of information about Ebola and what source to you trust the most? (List and rank if more than one)	
Systems affected by outbreak	Can you please list, to the best of your knowledge, which aspects of the mining operations were most affected during the outbreak? (List and rank)	Production Mining capacity Human Resources H&S
	Why do you think this was the most affected?	
CLD	Please comment on the CLD diagram(s) that the research team have developed.	Discussion

CLD, causal loop diagram.
